# Circular RNA circ_0068464 combined with microRNA-383 regulates Wnt/β-catenin pathway to promote the progression of colorectal cancer

**DOI:** 10.1080/21655979.2022.2036905

**Published:** 2022-02-15

**Authors:** Yan Zou, Limin Liu, Jie Meng, Meiyu Dai

**Affiliations:** Medical Science Laboratory, The Fourth Affiliated Hospital of Guangxi Medical University, Guangxi, China

**Keywords:** Colorectal cancer, hsa_circ_0068464, miR-383, Wnt/β-catenin pathway

## Abstract

This study was to clarify the influence and mechanism of circular RNA hsa_circ_0068464 (circ_0068464) on the development of colorectal cancer (CRC). First, we combined bioinformatics analysis and the high-throughput sequencing to determine the expression profile of circRNAs in CRC dataset, and screened out the differentially expressed circ_0068464. Subsequently, qRT-PCR was utilized to measure circ_0068464 expression in CRC and normal cancer-adjacent tissues, CRC cell lines (SW480, SW620, HT29, LS174T and HCT116) and human fetal intestinal epithelial cell (FHC). The results revealed that circ_0068464 was abnormally up-regulated in CRC cells and tissues. Knockdown of circ_0068464 could inhibit CRC cell migration and proliferation and promoted apoptosis while suppressing the expression of Wnt/β-catenin pathway-related proteins (β-catenin, cyclin D1, C-myc and LEF-1). In addition, tumorigenic assays in nude mice confirmed that circ_0068464 downregulation significantly inhibited tumor growth and lung metastasis. Further, the binding interaction between circ_0068464 and microRNA-383 (miR-383) was verified by dual-luciferase assay and RNA immunoprecipitation assay. And miR-383 was significantly down-regulated in CRC tissues and cells. Interfering with miR-383 expression reversed the inhibitory effect of circ_0068464 knockdown on CRC cells. In conclusion, circ_0068464 targets miR-383 to regulate Wnt/β-catenin pathway activation, thereby promoting the development of CRC.

## Graphical Abstract

**Figure:**
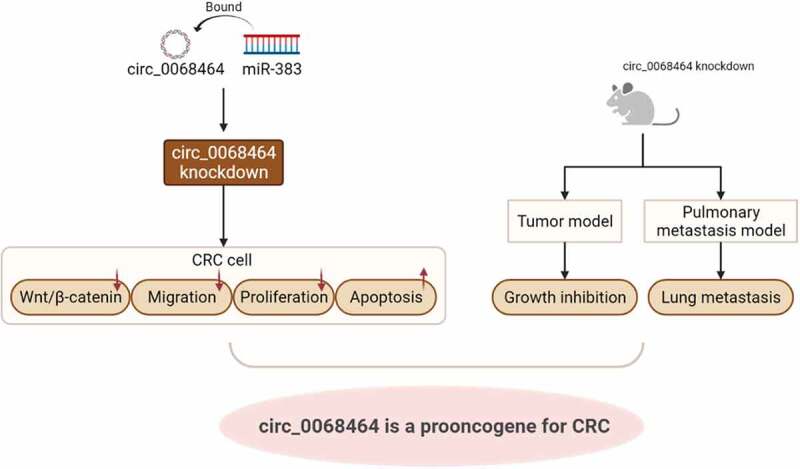

## 1. Introduction

As the third most known cancer, colorectal cancer (CRC) is the leading cause of cancer-related mortality across the world [1]. Annually, among deaths caused by cancers and cancer-related diseases all over the world, CRC accounts for about 10% [2]. With lasting development in developing nations, by 2035, the number of patients with CRC is predicted to increase to 25 million new cases worldwide [3]. CRC is a disease without characteristic symptoms before it reaches an advanced stage, so the early diagnosis of CRC in symptomatic patients remains a problem [4]. In addition, some studies have pointed out that genetics, lifestyle, obesity and environmental conditions may all be the possible factors for the occurrence and development of CRC. Nevertheless, the exact mechanism of the occurrence of CRC is not fully understood [5]. At present, the therapy of CRC in clinical application mainly includes surgical, radiational and drug adjuvant therapy [6]. However, the surgery for rectal cancer is very complicated. Robot-assisted laparoscopic total mesorectal excision and transanal minimally invasive surgery for total mesorectal excision are the main surgical techniques. However, both techniques, which are relatively uncommon, demand highly professional knowledge and reliable studies. Chemoradiotherapy, with unsatisfactory treatment effects, is prone to cause some side effects, such as cytotoxicity and neurotoxicity. In addition, the diagnosis and treatment of CRC are difficult because of the recurrence and metastasis of CRC tumors, particularly lung metastasis, resulting in a poor prognosis for patients [7]. Therefore, it is urgent to find new therapeutic targets, potential biomarkers and strategies for the diagnosis and treatment of CRC.

Circular RNA (circRNA), a novel RNA, is a special class of long non-coding endogenous RNA molecules [8]. CircRNA can regulate gene expression either transcriptionally or post-transcriptionally. CircRNAs exert a regulatory function by combining with microRNAs (miRNAs) or other molecules [9]. An increasing proof has verified that circRNAs are included in the development and progression of CRC [10,11]. Some studies have reported that circ_PRKDC promotes the development of CRC with the miR-198/DDR1 regulatory axis [12]. Circ_0005576 can promote the deterioration of CRC with miR-874/CDK8 axis [13]. By whole transcriptome sequencing analysis, some scholars found that 10 circRNAs were abnormally expressed in CRC. Among 10 abnormal circRNAs, the expression of 6 circRNAs was significantly raised while the expression of the other 4 was significantly fallen; and circRNA-miRNA-mRNA regulatory networks in CRC were built [14]. The result of whole transcriptome sequencing analysis suggested that circRNAs own the potentiality to be applied as biomarkers to predict the progression and prognosis of diseases. Although the understanding of circRNAs is improved, the potential correlation between circRNAs and the progression of CRC has not been fully elucidated. Circ_0068464 is a newly discovered circRNA, and its association with CRC has not been reported yet, so we tried to reveal the signaling network of circ_0068464 in the regulation of CRC progression.

CircRNAs can act as a competitive endogenous RNA (ceRNA) that binds miRNAs and negatively regulates their activity through miRNA response elements (MREs) [15]. MiRNAs are a group of evolutionarily conservative short non-coding RNAs that are verified to be included in various biological functions [16]. A lot of researches have disclosed that miRNA expression is abnormal in a series of tumors. MiRNAs can be involved in tumorigenesis by mediating gene expression or activating signaling pathway [17]. Some studies have indicated that miR-383-5p expression is low in the serum of CRC patients, and it is up-regulation can facilitate the efficacy and prognosis of neoadjuvant chemotherapy in CRC [18]. Besides, miR-383 can suppress the occurrence of CRC through the regulation of the expression of CREPT/RPRD1B in CRC [19]. However, the molecular mechanism of miR-383 in CRC has not been fully elucidated, and the association of miR-383 with circ_0068464 in CRC is unknown.

Improperly activating proto-oncoprotein β-catenin is regarded as the reason for inducing tumor formation. The activation of β-catenin causes the occurrence of nuclear accumulation, thereby promoting the combination between β-catenin and T-cell transcription factors, as well as the up-regulation of proto-oncogenes, including Axin2, c-myc, and cyclinD1 [20]. Abnormal Wnt/β-catenin signaling has been frequently discovered in various cancers, especially in CRC. The signaling cascade is the core to carcinogenesis [21]. The activation of abnormal Wnt/β-catenin signaling is the marker of poor prognosis in CRC patients [22]. Therefore, Wnt/β-catenin signaling owns potential significance for the target in the therapy of CRC.

Based on the above studies, we speculated that circ_0068464 might act as a ceRNA binding miR-383 to regulate the progression of CRC. Therefore, in this study, we determined the expression of circ_0068464 and miR-383 in CRC, and investigated the role of circ_0068464 in CRC and its molecular mechanism through in vivo and in vitro experiments to find new biomarkers and targets of action for the diagnosis and treatment of clinical CRC.

## 2. Materials and methods

### 2.1 Bioinformatic analysis

CRC-related circRNA datasets including GSE138589, GSE142837 and GSE126094 were from the Gene Expression Omnibus (GEO) database (https://www.ncbi.nlm.nih.gov/gds/?term=). And circRNAs in SW480 cells were detected using DNA arrays (DNA microarray, Affymetrix, USA). Wayne diagrams were plotted to screen for up-regulated circRNAs expression.

### 2.2 Clinical samples

CRC and normal cancer-adjacent tissues were collected in 60 CRC patients who received treatment from June 2018 to December 2020 in our hospital. The collection of tissue samples was checked with histopathological examination and identified independently by three professors of pathology. The Research Ethics Committee of our hospital approved this study (Approval number: KY2019212), and informed consent forms were signed by all subjects.

### 2.3 Cell culture

Normal colonic epithelial cell line (FHC) and CRC cell lines (HCT116, HT29, SW480, SW620, and LS174T) were provided from American Type Culture Collection (ATCC, USA). All the cells were cultured in Dulbecco’s modified eagle medium (DMEM) or 1640 medium with 10% fetal bovine serum (FBS, Gibco, USA), 10 mg/mL streptomycin and 100 mg/mL penicillin (Gibco). Then, these cells were placed in an incubator (Thermo, USA) containing 5% CO_2_ at 37°C.

### 2.4 Cell transfection

Circ_0068464 small interfering RNA (si-circ_0068464) and negative control (siNC), miR-383 inhibitor and negative control (NC inhibitor), miR-383 mimics and negative control (NC mimics) were synthesized by Guangzhou RiboBio Co., Ltd. (China). When HCT116 and LS174T cells were cultured to 60–70% confluence, the above RNA was transfected into cells using Lipofectamine 2000 (Invitrogen, USA). After 6-h culture, the complete medium was replaced, and the cells were collected after another 48-h culture.

### 2.5 Tumor xenograft test in nude mice

The Animal Center of Fourth Affiliated Hospital of Guangxi Medical University offered 24 SPF BALB/c male nude mice (weight: 18–22 g; age: 4–6 weeks). Twelve nude mice were separated randomly into two groups (n = 6): siNC group and si-circ_0068464 group. Further, HCT116 cells transfected negative siRNA and circ_0068464 siRNA were re-suspended, respectively. Then, the cells were digested to 1 × 10^7^ cells/ml. Subsequently, the right axilla of nude mice was injected subcutaneously into 200 μL cell suspension. The size of the tumor was determined every 3 days since subcutaneous injection. The formula ‘Volume = 0.5× length × width^2^’ was chosen to calculate tumor size [23].

Twelve nude mice were selected into two groups randomly (n = 6): NC group and si-circ_0068464#1 group. Negative siRNA and circ_0068464 siRNA#1 were transfected into HCT116 cells. Then, after digestion and resuspension of these HCT116 cells, the tail veins of nude mice were injected into 2 × 10^6^ cells [24]. Mice were killed, and their lungs were extracted after 30 days.

### 2.6 Quantitative real-time polymerase chain reaction (qRT-PCR)

TPizol (Thermo, USA) was applied to obtain total RNA in the tissues and cells. Further, reverse transcription kit (Thermo, USA) was utilized for the preparation of cDNA. On the basis of the instruction of SYBR GREEN kit (TaKaRa, Japan), the expression of circ_0068464, miR-383 and EIF4A2 was detected, with GAPDH and U6 as internal reference control. Six replicates were set up for the experiment. The relative expression of the target gene was calculated using 2^−ΔΔCt^ method. Table 1 shows the primer sequences.

**Table 1.** Primer sequences

RNA	Sequences (5’ to 3’)
circ_0068464	F: 5’-CAGAGACGATTGGCTGGTGA-3ʹ
	R: 5’-TGATAAATTGCCCAACAAAGAGACT-3ʹ
miR-383	F: 5’- GATCAGAAGGTGACTGTGG-3ʹ
	R: 5’- GAACATGTCTGCGTATCTC-3ʹ
EIF4A2	F: 5’- CTCTCCTTCGTGGCATCTATGC-3ʹ
	R: 5’- TGGCTGTCTTGCCAGTACCTGA-3ʹ
GAPDH	F: 5’- GTCTCCTCTGACTTCAACAGCG-3ʹ
	R: 5’- ACCACCCTGTTGCTGTAGCCAA-3ʹ
U6	F: 5’ – CTCGCTTCGGCAGCACAT-3ʹ
	R: 5’- TTTGCGTGTCATCCTTGCG-3ʹ

### 2.7 RNA fluorescence in situ hybridization (FISH)

HCT116 cells were fixed on 0.4% Poly-L-lysine-coated coverslips, and then paraformaldehyde was adopted to fix the cells for 10 min. After that, 10-min incubation was conducted with 0.5% TritonX-100. After the treatment of proteinase K (2 μg/mL), glycine, and ethylphthalating reagent, 250 μL of prehybridization solution was placed into the cells for another 1-h incubation at 42°C. Subsequently, the prehybridization solution was aspirated. Hybridization was conducted at 42°C for a whole night after adding 250 μL hybridization solution with Alexa488-labeled circ_0068464 probe (300 ng/mL). Then, PBST was applied to wash the cells for three times, and nuclei of cells were stained with DAPI (1:800) staining solution diluted in PBST. Further, a 24-well plate was carried out to place the cells for 5-min staining. Subsequently, PBST was performed to wash the cells for 3 times (3 min per time). The cells were then mounted with an antifade mounting medium, and the expression and location were detected in HCT116 cells using confocal microscopy.

### 2.8 RNaseR treatment

RNA in HCT116 and LS174T cells was obtained and split into two parts: one part used RNase R (Epicenter Technologies, USA) to digest and degraded linear mRNA; another part was applied as the control of digestion buffer. After both parts were incubated at 37°C for 30 min, the expression of linear 0068464 and circ_0068464 was determined using qRT-PCR [25].

### 2.9 MTT

After transfection, a 96-well plate was adopted to seed cells in the logarithmic growth phase at a density of 10,000 cells/well. Then, the cells were, respectively, cultured for 24 h, 48 h, and 72 h. Further, cells in each group were placed into 20 μL MTT solution (5 mg/mL) for 4 h. Subsequently, the culture supernatant was aspirated, and 150 μL of DMSO was added, then the cells were shaken for 15 min. The absorbance value was measured when the wavelength was at 490 nm in a microplate reader.

### 2.10 Flow cytometry

Trypsin was adopted to digest cells into centrifuge tubes. Then, precooled sterile PBS was carried out to rinse the cells for twice, and the concentration of cells was set to 1 × 10^5^ cells/mL. Subsequently, 200 μL cell suspension was collected. After 10 μL Annexin V-FITC was added, 10 μL PI solution was put into the cell suspension. Furthermore, 10-min incubation was performed for the cell suspension at ambient temperature in the dark. Then, 500 μL PBS was added. Flow cytometry was carried out to determine apoptosis.

For cell cycle assay, a 6-well plate was performed to seed cells in the logarithmic growth phase. In a serum-free medium, 24-h culture was conducted until the density of the cells was appropriate. After the cells were collected, 70% pre-cooled ethanol was utilized to fix the cells at 4°C overnight. After centrifugation, 400 μL RNAase solution and PI staining solution were added to the cells. Cells were stained for 30 min at room temperature in the dark. All results were detected and analyzed with flow cytometry.

### 2.11 Transwell assay

Transwell was utilized to determine invasion and migration of cells. Specifically, for cell migration, 100 μL cell suspension was poured into the upper chamber, and 700 μL culture medium with 20% FBS was placed into the lower chamber. After being incubated at 37°C with 5% CO_2_ for 12–24 hours, the transwell inserts were collected. PBS was adopted to wash the transwell inserts. After drying, a total of 6–10 views fields were chose to be observed through upright microscope. The number of positive cells in each view field was recorded, and then three view fields were selected randomly to photograph and statistically analyze.

For cell invasion, matrigel was melted at 4°C, then was extended onto the upper chamber. Subsequently, 100 μL cell suspension was put into the upper chamber, while the lower chamber was put into 700 μL culture medium with 20% FBS. Subsequent manipulations were consistent with the migration assay above.

### 2.12 Western blot

Total protein was extracted with RIPA lysing solution (Gibco, USA). BCA kit (Thermo, USA) was applied to detect the concentration of proteins. After that, 20 μg protein was separated with 10% SDS-PAGE and then transferred to the polyvinylidene difluoride (PVDF) membrane. Subsequently, the membrane was blocked with 5% nonfat dry milk for 1 h at room temperature, and then incubated with the primary antibodies at 4°C overnight. Secondary antibodies were added for another one-hour incubation at room temperature after the membrane was washed for three times. The membrane was then washed three times, and a chemiluminescence reagent was put to reveal the protein. Gel imaging system was adopted to collect images. Image J software was adopted for the analysis of the grayscale of the protein bands. GAPDH was adopted as an internal control to calculate the relative protein expression values

### 2.13 H&E staining

Lung tissues of nude mice were fixed with 4% paraformaldehyde. Paraffin-embedded sections (5 μm) were dewaxed in xylene I and II, respectively. After deparaffinization, 100%, 95%, 85%, and 75% gradient ethanol were successively placed for 5 min in each slice to deparaffinize into water. Then, distilled water was performed to wash the slices. After being stained with hematoxylin-eosin, the slices were successively put in 95% ethanol and absolute ethanol I and II for 5 min each and then were cleared with xylene I and II. Subsequently, the slices were taken out and dried. Neutral gum was utilized to seal the slices. Tumor nodules in lung tissues were observed with a microscope. The number of tumor nodules was quantitatively analyzed.

### 2.14 Dual-luciferase reporter assay

Wild-type circ_0068464 (circ_0068464-WT) and mutant (circ_0068464-MUT) plasmids were constructed by Promega Corporation, USA. The corresponding plasmids, miR-383 mimics and NC mimics were transfected, respectively, into HCT116 and LS174T through Lipofectamine 2000. After 48 h transfection, on the basis of the instructions of a dual-luciferase reporter assay kit, luciferase activity was detected.

### 2.15 RNA immunoprecipitation (RIP)

Magna RIP RNA-binding protein immunoprecipitation kit (Sigma, USA) was adopted to conduct RIP. First, 1.5 × 10^7^ HCT116 or LS174T cells were added with 0.25 μL protease inhibitors, 0.125 μL RNase inhibitors and 50 μL LRIP lysis buffer for the preparation of cell lysates. After the cells were centrifuged, the supernatant was obtained and then incubated with RIP wash buffer following manufacturer’s instructions. Finally, mRNA levels in RNA-binding protein complexes treated by magnetic bead adsorption were measured through qRT-PCR.

### 2.16 Statistical analysis

SPSS 22.0 software was adopted for one-way analysis of variance (ANOVA) and independent samples t-test analysis for normally distributed data. All data were presented as mean ± standard deviation (SD). Pearson correlation analysis was performed for the analysis of the association between circ_0068464 and miR-383 expression in CRC tissues. *P* < 0.05 was considered to be statistically significant.

## 3 Results

### 3.1 Circ_0068464 was upregulated in colorectal cancer tissues and cells

**Figure 1.**
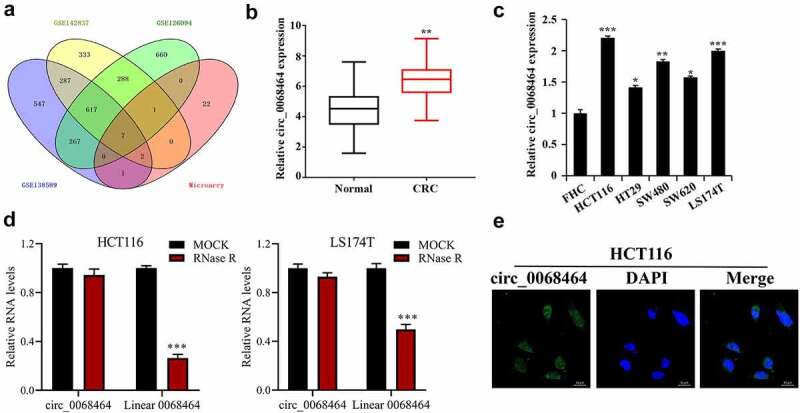
Circ_0068464 expressions in colorectal cancer tissues and cells. (a) GEO database and the intersection of DNA arrays were utilized to explore CRC-related circRNA; (b–c) QRT-PCR was applied to determine circ_0068464 expression in CRC tissues (b), ** *P* < 0.01 vs. Normal group) and in CRC cell line (C, *P < 0.05 vs. FHC group); (d) RNaseR digestion was used to verify the ring structure of circ_0068464 in HCT116 and LS174T cells, ***P < 0.001 vs. Mock group; (e) FISH experiments were performed to detect the subcellular localization of circ_0068464.

To explore CRC-related crucial circRNA, three data sets from GEO database were intersected with the results of DNA arrays. Among the results of intersection, seven circRNAs met conditions were collected: hsa_circ_0000518, hsa_circ_0072088, hsa_circ_0005273, hsa_circ_0000517, hsa_circ_0048104, hsa_circ_0067301 and hsa_circ_0068464 (Figure 1a). To further check that circ_0068464 in CRC tissues was up-regulated, CRC tissues and normal cancer-adjacent tissues were determined, and the result indicated that circ_0068464 expression in CRC tissues was up-regulated notably (Figure 1b). According to the result of further detection in CRC cell lines, circ_0068464 expression in CRC cell lines was increased significantly, and was the highest in HCT116 and LS174T cells (Figure 1c). Then, in order to determine that circ_0068464 belongs to circRNA, the expression of circ_0068464 and linear 0068464 was detected after RNaseR treatment. The result of detection revealed that circ_0068464 was resistant to RNaseR digestion compared to the linear 0068464 (Figure 1d). According to the result of FISH, circ_0068464 was mainly localized in the cytoplasm of CRC cells (Figure 1e). All the results disclosed that circ_0068464 was a circRNA and was involved in the occurrence and development of CRC.

### 3.2 Knockdown of circ_0068464 can inhibit malignant phenotype of colorectal cancer cells

**Figure 2.**
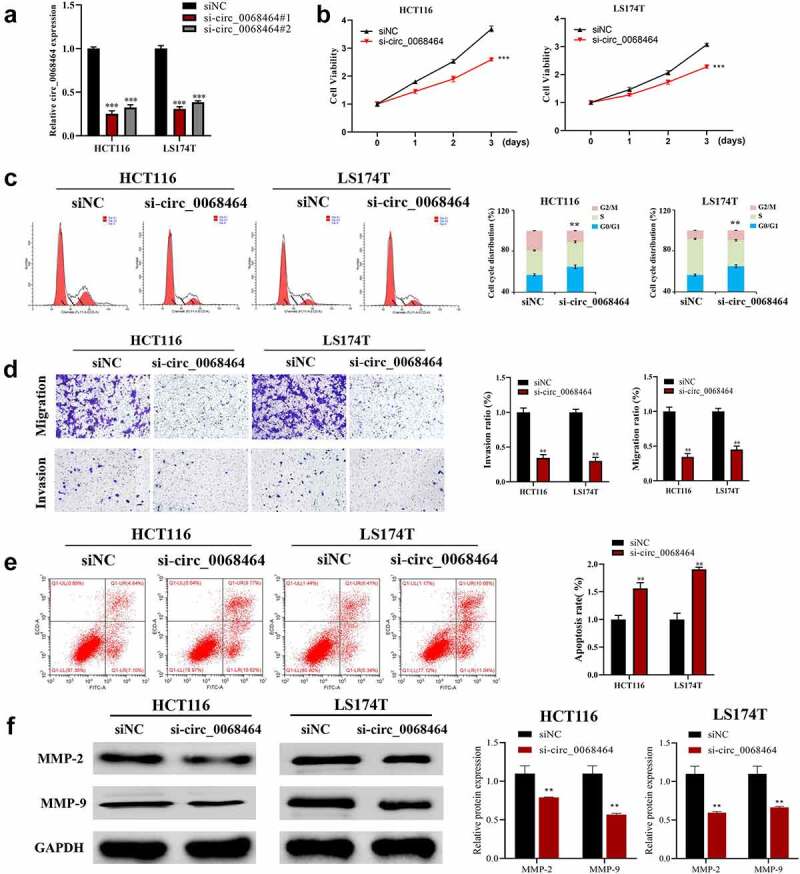
Effects of knockdown of circ_0068464 on malignant phenotype of colorectal cancer cells. (a) The expression of circ_0068464 in the cells in each group was detect using qRT-PCR. For HCT116 or LS174T cells, (b) MTT was utilized to measure proliferation rate; (c-e) flow cytometry was carried out to check the cycle (c) and apoptosis rate (e); (d) transwell was adopted to determine the abilities of migration; (f) Western blot was adopted to check MMP-2 and MMP-9 expression. **P < 0.01 vs. siNC group.

To investigate the effect of circ_0068464 on the occurrence and development of CRC, the expression of circ_0068464 was knocked down. According to the result of qRT-PCR, both si-circ_0068464#1 and #2 could decline the expression of circ_0068464, and the effect of si-circ_0068464#1 was better (Figure 2a). The result of qRT-PCR revealed that knockdown of circ_0068464 was successful. Subsequently, MTT, flow cytometry, and Transwell were utilized to detect whether circ_0068464 could affect malignant phenotype of CRC cells. The result of detection revealed that compared with siNC group, knockdown of circ_0068464 could suppress the proliferation, invasion, and migration of cells significantly; besides, the up-regulation of cells in G0/G1 phase indicated a delay in cell division and an arrest in the cell cycle (Figure 2b–d). Furthermore, compared with siNC group, knockdown of circ_0068464 could improve apoptosis significantly (Figure 2e). And knockdown of circ_0068464 inhibited MMP-2 and MMP-9 expression (proteins that were closely related to invasion) in cells (Figure 2f). All results above indicated that, in CRC cells, malignant phenotype could be inhibited by knockdown of circ_0068464.

### 3.3 Circ_0068464 can target the expression of miR-383 in colorectal cancer

**Figure 3.**
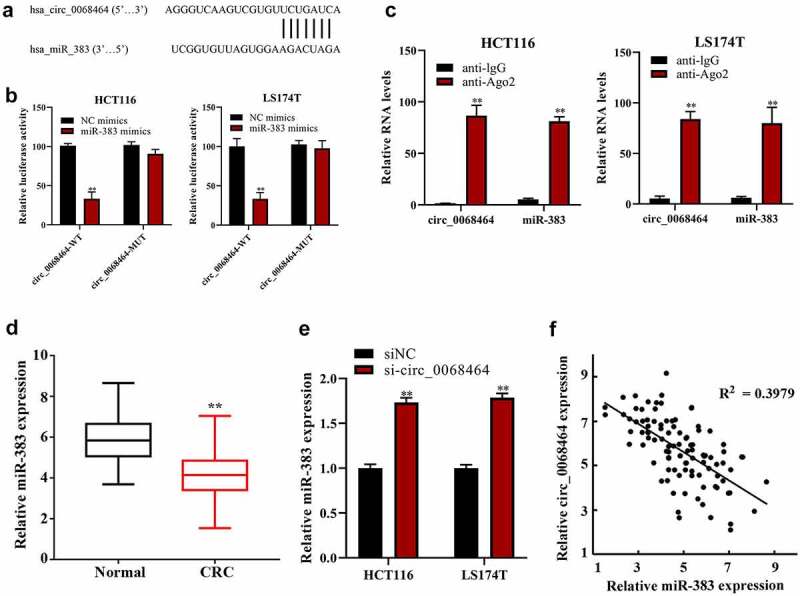
The correction between circ_0068464 and miR-383 in colorectal cancer. (a) The binding site between miR-383 and circ_0068464 was predicted by bioinformatics website; (b–c) dual-luciferase (b) and RIP (c) were applied to determine the targeted relationship between circ_0068464 and miR-383, **P < 0.01 vs. anti-IgG group; (d-e) QRT-PCR was utilized to check miR-383 expression in colorectal cancer tissues (d) and cells of each group (e), **P < 0.01 vs. Normal group; **P < 0.01 vs. siNC group; **P < 0.01 vs. NC group; (f) Pearson correlation analysis was utilized check the expression of correlation between circ_0068464 and miR-383 in colorectal cancer tissues.

To check the effect and mechanism of circ_0068464 on CRC, ENCORI database (http://starbase.sysu.edu.cn/) was adopted to predict and reveal the combination between circ_0068464 and miR-383 (Figure 3a). The result of dual-luciferase reporter gene experiments disclosed that there was a targeting relationship between circ_0068464 and miR-383 (Figure 3b). Further, the result of RIP experiment revealed that circ_0068464 and miR-383 were bond to each other (Figure 3c). And miR-383 expression in CRC group was reduced compared with normal group (Figure 3d). After the knockdown of circ_0068464, miR-383 expression in normal group was notably higher than that in siNC group (Figure 3e). According to the Pearson correlation analysis, circ_0068464 and miR-383 expression were correlated negatively (figure 3f). All the results above suggested that there was a targeting relationship between miR-383 in CRC and circ_0068464.

### 3.4 Knockdown of miR-383 can reverse the inhibitory effect of the knockdown of circ_0068464 on the progression of colorectal cancer

**Figure 4.**
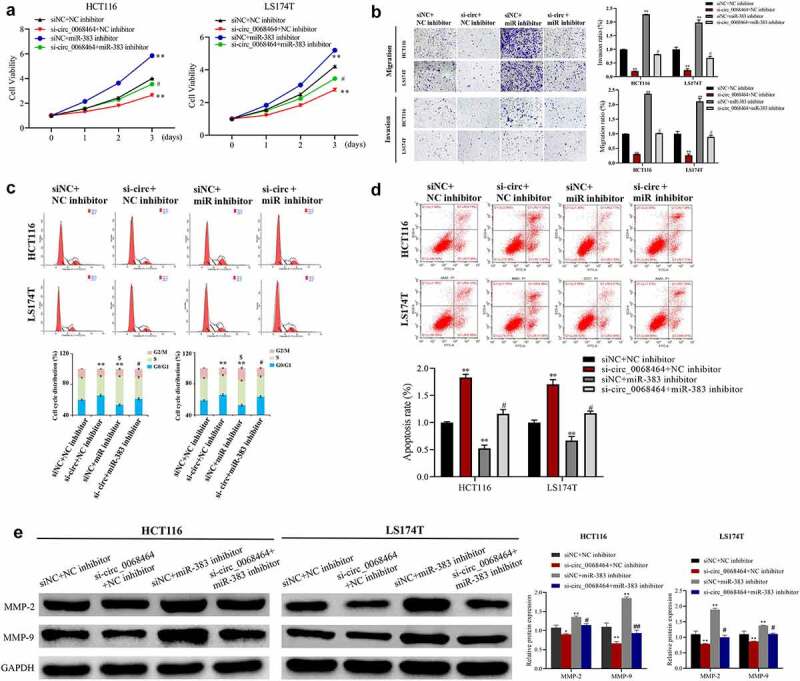
Effects of knockdown of miR-383 on circ_0068464 in colorectal cancer cells. (a) MTT was utilized to determine the proliferation rate of LS174T or HCT116 cells; (b) Transwell assay was applied to check the migration of HCT116 or LS174T cells; (c–d) flow cytometry was applied to check the cycle (c) and apoptosis (d) of HCT116 or LS174T cells; (e) Western blot was utilized to determine MMP-2 and MMP-9 expression in HCT116 or LS174T cells. **P < 0.01 vs. siNC+NC inhibitor group, ^##^ *P* < 0.01 vs. si-circ_0068464+ NC inhibitor group.

To explore whether miR-383 was involved in the effect of circ_0068464 on the progression of CRC, miR-383 expression was knocked down on the basis that HCT116 cell and LS174T cell inhibited circ_0068464. The result indicated that knockdown of circ_0068464 inhibited the migration, proliferation, and invasion of CRC cells, blocked the cell cycle, and promoted apoptosis. Compared with siNC+NC inhibitor group, knockdown of miR-383 could promote the proliferation, migration, and invasion of CRC cells and inhibit apoptosis; the correlation between cells in S phase and cellular fission was positive. However, on the basis of knockdown of circ_0068464, the effect of knockdown of circ_0068464 was reversed in si-circ_0068464+ miR-383 inhibitor group (Figure 4a–d). According to the result of Western blot, compared with the si-circ_0068464+ NC inhibitor group, MMP-2 and MMP-9 protein expression were significantly upregulated in the si-circ_0068464+ miR-383 inhibitor group (Figure 4e). All results indicated that miR-383 was an intermediary of circ_0068464 to exert a function in CRC.

### 3.5 Knockdown of circ_0068464 can target miR-383 to affect Wnt/β-catenin pathway activation to inhibit the growth of colorectal cancer

**Figure 5.**
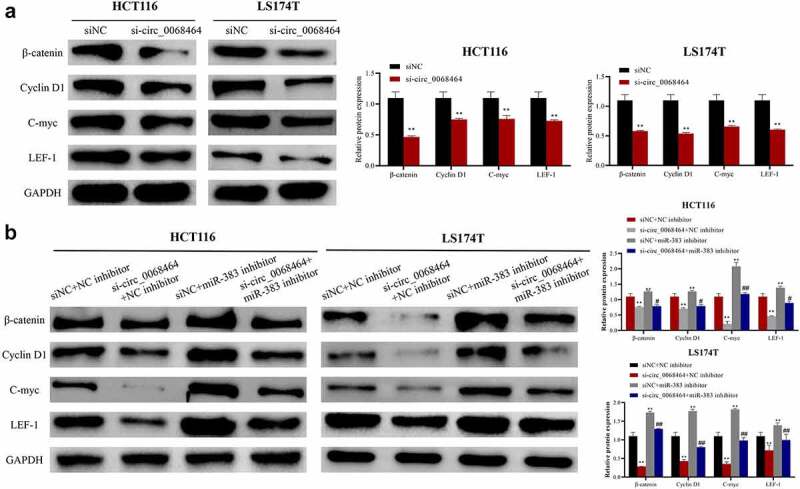
Effects of knockdown of circ_0068464 and miR-383 on Wnt/β-catenin pathway. (a) Western blot was utilized to detect the protein expression of LEF-1, Cyclin D1, C-myc and β-catenin in HCT116 or LS174T cells after knockdown of circ_0068464, **P < 0.01 vs. siNC group; (b) Western blot was applied to check β-catenin, Cyclin D1, C-myc and LEF-1 protein expression in HCT116 or LS174T cells in each group, **P < 0.01 vs. siNC+NC inhibitor group, ^##^*P* < 0.01 vs. si-circ_0068464+ NC inhibitor group.

To investigate the mechanism of circ_0068464, Wnt/β-catenin pathway-related protein expression in HCT116 and LS174T cells in each group was detected. According to the result, compared with siNC group, knockdown of circ_0068464 could inhibit β-catenin and downstream protein CyclinD1, C-myc and LEF-1 expression in cells, and could inhibit the activation of Wnt/β-catenin pathway (Figure 5a). Knockdown of miR-383 could increase Cyclin D1, β-catenin, C-myc and LEF-1 expression. Compared with si-circ_0068464+ NC inhibitor group, the inhibitory effect on the Wnt/β-catenin pathway was notably reversed in si-circ_0068464+ miR-383 inhibitor group (Figure 5b). All results above indicated that circ_0068464 targeted miR-383 to regulate the activity for Wnt/β-catenin pathway to exert biological functions.

### 3.6 *Knockdown of circ_0068464 can suppress the development and lung metastasis of colorectal cancer* in vivo

**Figure 6.**
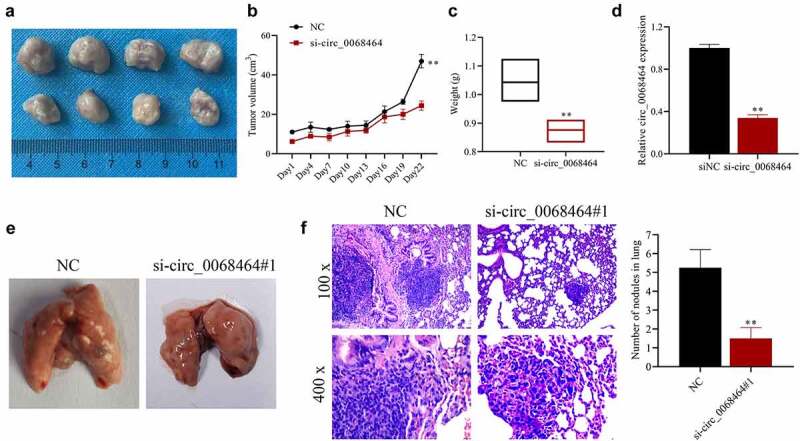
Knockdown of circ_0068464 can suppress the development and lung metastasis of colorectal cancer in vivo. (a) The images (top, NC group; down, si-circ_0068464 group) of tumor tissues of mice; (b–c) volume (b) and weight (c) in tumor tissues of mice in each group; (d) Circ_0068464 expression in tumor tissues of mice in each group was determined using qRT-PCR; (e) the illustrative diagram of lung tissues of mice in each group; (f, h and e) staining was utilized to observe tumor nodules in lung tissues. **P < 0.01 vs. siNC group.

To further verify the effect of circ_0068464 on the development of CRC in vivo, nude mouse tumor model and lung metastasis model were established. The result indicated that, compared with siNC group, knockdown of circ_0068464 could reduce the volume and weight of the tumor (Figure 6a–c). And after knockdown of circ_0068464, the expression of circ_0068464 in tumor was significantly reduced (Figure 6d), indicating the success of the establishment of nude mouse tumor model. Besides, in lung metastasis model, compared with siNC group, after knockdown of circ_0068464, metastatic lesion in lungs were reduced, and the quantity of metastatic nodules was declined (Figure 6e–f). All results above suggested that knockdown of circ_0068464 could suppress the development and lung metastasis of CRC in vivo.

## 4. Discussion

CRC, one of the most known malignant tumors worldwide, has so far been relatively difficult to diagnose and treat [26]. Studies have shown that thousands of circRNAs in the human peripheral blood have been detected by RNA sequencing [27], indicating that circRNAs have potential value for human disease diagnosis. Increasing studies have shown that the abnormal circRNAs expression exerts a crucial regulatory function in tumors, such as glioma [28], hepatocellular carcinoma [29], and gastric cancer [30]. In recent years, many studies have been devoted to finding biomarkers for CRC. Through bioinformatics analysis and high-throughput sequencing, some scholars have discovered that circRNA_0001178 and circRNA_0000826 are possible biomarkers of CRC liver metastasis [31]. In this study, through GEO database and DNA arrays, we found that circ_0068464 was aberrantly expressed in CRC. According to the further investigation, circ_0068464 expression was increased in CRC cells and tissues, and knockdown of circ_0068464 could significantly inhibit the malignant phenotype of CRC cells and the development of tumor in vivo. The result suggested that circ_0068464 can be used as a carcinogenic factor of CRC. However, there is no investigation on the effect of circ_0068464 in the tumors.

It has been shown that matrix metalloproteinases (MMPs), especially collagenase Type IV MMP-9, affect a lot in the metastasis and invasion of CRC [32]. As this study showed, knockdown of circ_0068464 was also found to be able to significantly inhibit CRC cell invasion and migration, as well as the expression of MMP2 and MMP9 proteins. In vivo experiments also demonstrated that circ_0068464 knockdown inhibited tumorigenic lung metastasis. Dai et al. [33] also demonstrated in vivo and in vitro experiments that downregulation of circMMP1 inhibited the expression of MMP2 and MMP9, which in turn inhibited CRC metastasis.

Some studies have found that miR-383 in CRC is down-regulated and is related to tumor size, TNM stage and lymph node metastasis [18]. MiR-383 may act as a tumor-inhibiting factor for CRC by directly targeting PAX6 to suppress cell invasion and proliferation [34]. The results of these studies indicate that miR-383 can inhibit the occurrence and growth of CRC. In this paper, based on the results of dual-luciferase assay and bioinformatics prediction, circ_0068464 and miR-383 interacted with each other, and as tumor-inhibiting factor, the expression of miR-383 was declined in CRC. The downregulation of miR-383 reversed the inhibitory effect of circ_0068464 knockdown on CRC cell proliferation and metastasis. This suggests that circ_0068464 could target and inhibit miR-383 to promote the progression of CRC. This was similar to the findings of Li et al. [35], who found that circ_0136666 accelerated CRC progression by directly targeting miR-383.

It has been indicated that miR-383 can regulate cellular activity through mediates the Wnt/β-catenin signaling pathway [36]. Gu et al. [37] investigated the interaction between Wnt1 and miR-383 through the analysis of Western blotting and luciferase activity, suggesting that miR-383 could control the Wnt/β-catenin signaling pathway by Wnt1. In this research, knockdown of circ_0068464 could suppress Wnt/β-catenin signaling pathway activity, while knockdown of miR-383 could reverse the inhibitory effect of the knockdown of circ_0068464 on Wnt/β-catenin signaling pathway. All the results above indicated that circ_0068464 could affect Wnt/β-catenin signaling pathway activity by miR-383. However, further studies are needed regarding the target genes of miR-383 and how they specifically regulate the Wnt/β-catenin signaling pathway activity involved in the progression of CRC.

## 5. Conclusion

In summary, this study has demonstrated that circ_0068464 is a new oncogene for CRC, and circ_0068464 targets and suppresses miR-383 to regulate the Wnt/β-catenin signaling pathway, thereby exerting oncogenic functions. Therefore, circ_0068464 has great potential for the diagnosis and treatment of CRC and may be a new target for clinical therapy of CRC.

## Data Availability

The data sets used and analyzed during the current study are available from the corresponding author on reasonable request.

## Highlights

1. Circ_0068464 is up-regulated in colorectal cancer (CRC), and miR-383 is down-regulated in CRC.
2. Circ_0068464 knockdown inhibits CRC cell migration and proliferation, and promotes apoptosis.
3. Circ_0068464 targets miR-383 to regulate Wnt/β-catenin pathway activation.
4. Circ_0068464 downregulation inhibits tumor growth and lung metastasis in vivo.
